# RETRACTION: Oxidative Stress and Pulmonary Changes in Experimental Liver Cirrhosis

**DOI:** 10.1155/omcl/9849310

**Published:** 2025-12-17

**Authors:** Oxidative Medicine and Cellular Longevity

RETRACTION: R. Salatti Ferrari, D. Pase da Rosa, L. F. Forgiarini, S. Bona, A. S. Dias, and N. P. Marroni. “Oxidative Stress and Pulmonary Changes in Experimental Liver Cirrhosis,” *Oxidative Medicine and Cellular Longevity* 2012, no. 1 (2012): https://doi.org/10.1155/2012/486190.

The above article, published online on 17 December 2012 in Wiley Online Library (wileyonlinelibrary.com), has been retracted by agreement between the journal Editor‐in‐Chief, Jeannette Vasquez‐Vivar and John Wiley & Sons Ltd. The retraction has been agreed after an investigation into figure concerns raised by *Hydrophis melanocephalus* on PubPeer [1]. Specifically, duplicate bands and splicing were detected in Figure 9.

The authors agree with this decision.
